# A comparison between transoral endoscopic thyroidectomy vestibular approach and transareolar thyroidectomy regarding postoperative pain and cosmetic outcomes: A systematic review and meta-analysis

**DOI:** 10.12669/pjms.40.11.9712

**Published:** 2024-12

**Authors:** Hyder Osman Mirghani, Ibrahim Altedlawi Albalawi

**Affiliations:** 1 Hyder Osman Mirghani, Professor of Internal Medicine, Department of Internal Medicine, Faculty of Medicine, University of Tabuk, Tabuk, Saudi Arabia; 2 Ibrahim Altedlawi Albalawi, Professor of Surgical Oncology, Department of Surgery, Faculty of Medicine, University of Tabuk, Tabuk, Saudi Arabia

**Keywords:** Transoral thyroidectomy vestibular approach, Trans-areolar approach, Cosmetic outcomes, Postoperative pain score

## Abstract

**Objectives:**

Remote scarless surgeries have been developed for cosmetic reasons; literature showed that remote endoscopic surgeries were superior to conventional thyroidectomy. However, no researchers have compared the transoral thyroidectomy vestibular approach and transareolar approach regarding cosmetic outcomes and postoperative pain score. This meta-analysis assessed the same among patients with papillary microcarcinoma, thyroid disease, and small-size papillary carcinoma.

**Methods:**

We searched PubMed MEDLINE, Web of Science, SCOPUS, and Cochrane Library from the date of the first inception up to January 2024. The general keywords used are trans-areolar thyroidectomy, trans-oral thyroidectomy vestibular approach, scar-less thyroidectomy, remote thyroidectomy, pain score, and cosmetic effects. The MeSH terms “mouth”, “areola”, “nipples”, “vestibular”, “oral”, “endoscopes”, “endoscopic”, and “thyroid” were used. Out of the 389 studies and 114 remained after the removal of duplication, from them, 22 full texts were screened, and only 10 studies were included in the final meta-analysis.

**Results:**

The tansoral thyroidectomy vestibular approach showed better cosmetic outcomes compared to the trans-areolar approach, odd ratio, 1.26, 95% CI, 0.53-1.99. However, no significant statistical difference was found regarding postoperative pain score, odd ratio, -0.11, 95% CI, -0.26-0.04, and central lymph node dissection, odd ration, 1.52, 95% CI, -0.14-3.17.

**Conclusion:**

The transoral endoscopic thyroidectomy vestibular approach was better compared to the trans-areolar approach in terms of cosmetic outcomes, no differences were evident regarding pain score and central lymph node dissection. Further larger well-controlled studies assessing operative and postoperative outcomes are needed.

## INTRODUCTION

Thyroidectomy is on the rise due to the increasing indications (differentiated thyroid carcinoma and other thyroid disease).^1^ Open thyroidectomy is the gold standard for those undergoing thyroidectomy, however, the visible unsightly permanent neck scar is of great concern to the patients. The increasing demand for addressing cosmetic concerns leads to the development of remote minimally invasive thyroidectomies.^2^

Huscher et al.^3^ were the first to perform endoscopic thyroidectomy in the year 1997, since then, there has been a widespread use of remote scar-less surgeries in the past few decades. The transoral endoscopic vestibular approach is the most commonly used, other minimally invasive approaches include axilla-breast, trans-axillary, areolar, and subclavian. Despite being minimally invasive, some degree of scarring remains following the above novel techniques. In addition, there is an increasing awareness regarding the safety of these procedures due to the long flap dissection required.^4^,^5^ Previous studies showed the superiority of endoscopic thyroidectomies compared to conventional thyroidectomy in terms of the patient’s quality of life.^6^,^7^ The areolar approach (AA) thyroidectomy is among the most common methods for endoscopic and robotic-assisted thyroidectomy, the procedure showed good feasibility, surgical view, and minimal invasiveness.^8^

The transoral endoscopic thyroidectomy vestibular approach (TOETVA) is the only true scar-less thyroidectomy and requires minimal flap dissection, the procedure was developed in the year 2016.^9^ The procedure has gained popularity among patients and Surgeons due to its safety, short learning curve, and good cosmetic outcomes and quality of life.^10^,^11^ Literature comparing the transoral endoscopic thyroidectomy vestibular approach and areolar approach thyroidectomy is scarce.^1^,^12^ Therefore, this meta-analysis aimed to compare the transoral endoscopic thyroidectomy vestibular approach and areolar approach in terms of pain score and cosmetic outcomes.

## METHODS

### Eligibility criteria according to PICOS:

This meta-analysis was conducted during December 2023 and January 2024 with adherence to the PRISMA Guidelines.

### Characteristics of the study subjects:

This meta-analysis included 10 studies, all were conducted in China, the age of the patients ranged from 22.2±3.0 to 41.2±11.9 years, the majority were females (ranged from 50% to 100%), lobectomy was conducted in eight studies, while the patients underwent total thyroidectomy in two studies. The indication for surgery was unilateral papillary thyroid carcinoma in three studies, papillary thyroid microcarcinoma in another three studies, unspecified thyroid disease in two studies, benign thyroid nodules in one study, and papillary thyroid carcinomas <2cm in the reaming study.

### Ethical statement:

This study was exempted from the ethical clearance because it is a meta-analysis. In addition, the authors did not include any article published by them.

### Inclusion criteria:

Studies were eligible if they were randomized controlled trials, case-control studies, prospective, and retrospective studies on humans and comparing Trans areolar thyroidectomy, and transoral video-assisted thyroidectomy via vestibular approach.

### Exclusion criteria:

Case reports, case series, opinions, editorials, systematic reviews, and study protocols were excluded.

### Outcome measures:

The outcome measures were pain score and cosmetic outcomes.

### Literature search and data extraction:

**Fig.1:**
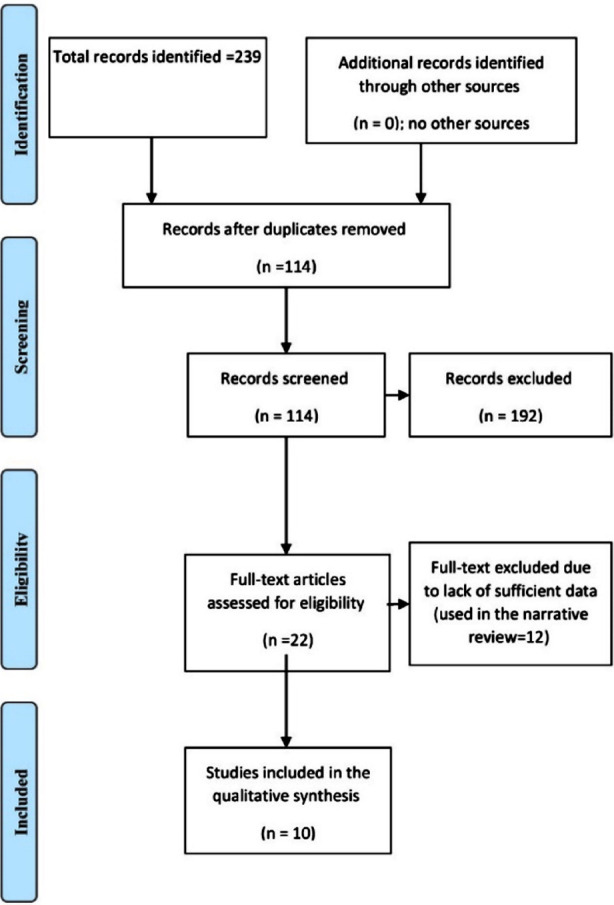
A literature search of transoral endoscopic vestibular approach thyroidectomy and areolar approach regarding postoperative pain and cosmetic outcomes.

**Fig.2:**
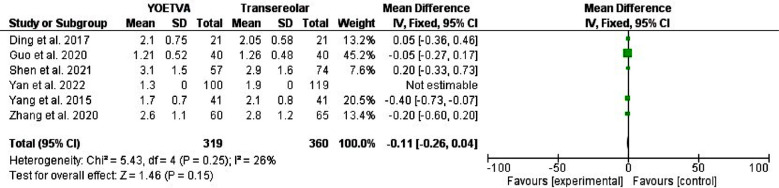
Postoperative pain among patients with transoral approach and areolar thyroidectomy.

**Fig.3:**
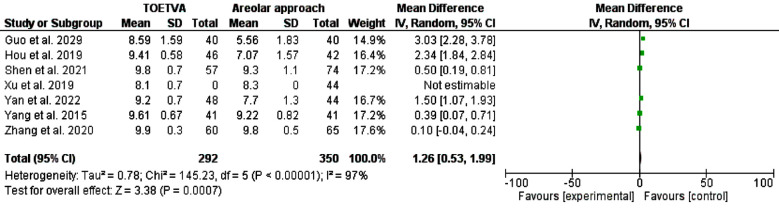
Cosmetic outcomes among patients with transoral approach and areolar thyroidectomy.

**Fig.4:**
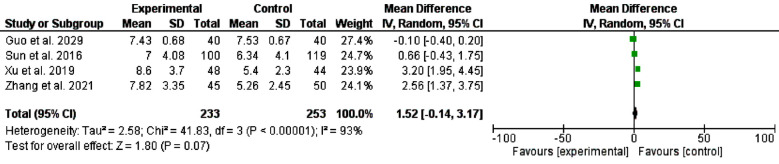
Central lymph node dissection feasibility among patients with transoral approach and areolar thyroidectomy.

A systematic literature search was carried out in PubMed MEDLINE, Web of Science, SCOPUS, and Cochrane Library from the date of the first inception up to January 2024. The two reviewers (H.M and I. A) searched the literature for relevant articles. The general keywords are trans-areolar thyroidectomy, trans-oral thyroidectomy vestibular approach, scarless thyroidectomy, remote thyroidectomy, pain score, and cosmetic effects. The MeSH terms “mouth”, “areola”, “nipples”, “vestibular”, “oral”, “endoscopes”, “endoscopic”, and “thyroid” were used. In addition, the titles, abstracts, and references of the included studies were screened. We identified 239 studies, 114 remained after the removal of duplication, and 22 full texts were screened. However, only ten studies were included in the final meta-analysis. A datasheet was used to extract the author’s name year and country of publication, age, sex, indication of surgery, pathology recovered, pain score, and cosmetic effects. Table-I and II and Fig.1-4.

**Table-I:** Basic characteristics of patients with transoral approach and areolar thyroidectomy.

Author	Country	Age/years	Females	Type of operation	Pathology
Ding et al.2017^14^	China	33.1 ±2.4 vs. 34.2 ±2.6	50% vs. 60%	thyroidectomy	Thyroid disease
Guo et al. 2020^15^	China	29.8±0.96 vs. 33.75±1.19	All women	lobectomy	Papillary thyroid microcarcinomas
Shen et al. 2021^12^	China	37.8±12.4 vs. 41.2±11.9	63.2% vs. 64.9%	lobectomy	Benign thyroid nodules
Sun et al. 2016^16^	China	29.65±6.57 vs. 34.59±7.69	86% vs. 86.5%	Lobectomy	Papillary thyroid carcinomas <2cm
Xu et al. 2019^1^	China	30.46 ±6.93 vs. 33.3 ±6.94	91.7% vs. 88.6%	lobectomy	Papillary thyroid microcarcinomas
Yan et al. 2022^17^	China	32.14±6.54 vs. 34.56±7.06	73.8% vs. 83.3%	lobectomy	Papillary thyroid microcarcinoma
Yang et al. 2015^18^	China	31.9±8.8 vs. 31.0±8.9	75.8% vs. 63.3%	thyroidectomy	Thyroid disease
Zhang et al. 2020^19^	China	22.2±3.0 vs. 23.7±3.8	91.7% vs. 100%	lobectomy	Unilateral papillary thyroid carcinoma
Zhang et al. 2021^20^	China	33.4±6.8 vs. 34.4±7.6	70.4% vs. 100%	lobectomy	Unilateral papillary thyroid carcinoma.
Hou et al. 2019^21^	China	32.54±7.59 vs. 33.83±8.10	78.9% vs. 60%	lobectomy	Unilateral papillary thyroid carcinoma.

**Table-II:** Pain score, cosmetic outcomes, and lymph nodes dissection among patients with remote thyroidectomy.

Character	Pain score		Cosmetic outcomes		Lymph node dissection	

Author	TOETVA	Transeraolar	TOETVA	Transeraolar	TOETVA	Transeraolar
Ding et al.2017^14^	2.1±0.75	2.05±0.58	Not assessed	Not assessed	Not assessed	Not assessed
Guo et al. 2020^15^	1.21±0.52	1.26±0.24	8.59±1.59	5.56±1.83	7.43±0.68	7.53±0.67
Shen et al. 2021^12^	3.1±1.5	2.9±1.6	9.8±0.7	9.3±1.1	Not assessed	Not assessed
Sun et al. 2016^16^	Not assessed	Not assessed	Not assessed	Not assessed	7.00±4.08	6.34±4.10
Xu et al. 2019^1^	Not assessed	Not assessed	9.2 ±0.7	7.7 ±1.3	8.6 ±3.7	5.4±2.3
Yan et al. 2022^17^	1.3	1.9	8.1	8.3	Not assessed	Not assessed
Yang et al. 2015^18^	1.7 ± 0.7	2.1 ± 0.8	9.61±0.67	9.22±0.82	Not assessed	Not assessed
Zhang et al. 2020^19^	Not assessed	Not assessed	9.9±0.3	9.8±0.5	Not assessed	Not assessed
Zhang et al. 2021^20^	2.6 ± 1.1	2.8 ± 1.2	Not assessed	Not assessed	7.82 ± 3.35	5.26 ± 2.45
Hou et al. 2019^21^	Not assessed	Not assessed	9.41±0.58	7.07±1.57	Not assessed	Not assessed

### Risk of bias assessment:

Newcastle Ottawa Scale assessed the quality of the included studies, all the studies were of good quality with a range of 7-8.^13^

### Statistical analysis:

The data were analyzed by the most recent version of the RevMan system. We included seventeen cohorts from ten studies. The dichotomous and continuous date data were entered manually and the fixed or random effects were applied depending on heterogeneity. A P-value of <0.05 was considered significant.

## RESULTS

### Characteristics of the included studies:

This meta-analysis included ten studies^1^,^12^,^14^-^21^ all the studies were published in China, two were prospective, and eight were retrospective cohorts. The majority were females and their ages ranged from 22-42 years. Indication for surgery was papillary carcinoma in seven studies, benign thyroid nodules in one study, while in two studies indications were unspecified thyroid disease. Table-I and II. Postoperative pain was not different between the transoral endoscopic thyroidectomy vestibular approach and areolar approach, odd ratio, -0.11, 95% CI, -0.26-0.04, the Chi-square was 5.43, and the P-value for overall effect, 0.15. The heterogeneity was not significant, I^2^=26%, and the P-value for heterogeneity, was 0.25.

Regarding cosmetic effects, TOETVA was better than the areolar approach, odd ratio, 1.26, 95% CI, 0.53-1.99, the Chi-square was 145.23, and the P-value for overall effect was 0.0007. A significant heterogeneity was found, I^2^=97%, and P-value for heterogeneity < 0.001. Fig.3 Lymph node dissection was not different between OOETVA and trans-areolar approach, odd ratio, 1.52, 95% CI, -0.14-3.17, the Chi-square was 2.58, and the P-value for overall effect was 0.07. A significant heterogeneity was found, I^2^=93%, and the P-value for heterogeneity < 0.001. Fig.4.

## DISCUSSION

Postoperative pain control is essential following thyroidectomy; less pain means improved quality of life and early return to work. In addition, low pain can avoid opioid addiction.^22^ Many interventions were found to alleviate or prevent pain ranging from anesthetic administration before intubation to music use.^23^ Comparing pain between different endoscopic procedures is essential, visual analog scale (VAS) for pain assessment varies greatly between different endoscopic procedures depending on the site of incision. The visual analog scale is high in the jaw and while brushing the teeth in the transoral vestibular approach procedure.^24^

Previous studies showed that TOETVA and areolar approach postoperative pain was not different from conventional thyroidectomy.^25^,^26^ However, few meta-analyses compared TOETVA and the areolar approach regarding postoperative pain. Xia et al. 27 included only five studies and found no difference between TOETVA and areolar thyroidectomy. Our meta-analyses included the larger up-to-date studies and found no difference in pain scores between TOETVA and areolar thyroidectomy, odd ratio, -0.11, 95% CI, -0.26-0.04. The few included studies by Xia and colleagues limited their study. In addition, the authors included the results of Liu et al.^26^ who compared trans-subclavian and the trans-areolar approaches. A pleasing esthetical scar is the main reason behind the development of different minimally invasive thyroidectomy techniques outside the neck.^28^

Previous literature showed the superiority of the remote thyroidectomy compared to the transcervical conventional approach.^29^ This is the first study to include the largest number of studies and showed the superiority of TOETVA compared to the areolar approach regarding cosmetic outcomes with insignificant heterogeneiety (I^2^=97%), odd ratio, 1.26, 95% CI, 0.53-1.99. Xia et al^27^ pooled studies comparing TOETVA, areolar, and subclavian approaches and found similar results. Importantly Xia and colleagues’ results were limited by the high heterogeneity (I^2^=97%) between the included studies.

The feasibility of central lymph node dissection depends on the working space, TOETVA showed superiority for central lymph node accessibility compared to the bilateral axilla-breast approach, and transaxillary approach.^30^ Wang et al.^31^ found comparable results of central lymph node dissection between TOETVA and conventional thyroidectomy. However, comparisons between TOETVA and areolar approaches are lacking. The present results showed no significant statistical difference between TOETVA and transareolar approach, odd ratio, 1.52, 95% CI, -0.14-3.17, the Chi-square was 2.58, and the P-value for overall effect was 0.07. The strength of this meta-analysis is that it included the largest number of studies. However, the significant heterogeneity in cosmetic effects (I^2^=97%), and lymph nodes dissection (I^2^=97%) comparisons limited the current findings.

### Limitations

Because of the above the results of this meta-analysis should be viewed in the face of high heterogeniety observed in the cosmetic outcomes and lymph node dissection results. in addition, all the included studies were published in Asia which is a big limitation of this meta-analysis.

## CONCLUSION

The transoral endoscopic thyroidectomy vestibular approach was better compared to the areolar approach in terms of cosmetic outcomes, no differences were evident regarding pain score and central lymph node dissection. Further larger well-controlled studies assessing operative and postoperative outcomes are needed.
